# Photonic Crystal Fiber Mach-Zehnder Interferometer for Refractive Index Sensing

**DOI:** 10.3390/s120302983

**Published:** 2012-03-02

**Authors:** Jian-Neng Wang, Jaw-Luen Tang

**Affiliations:** 1 Department of Construction Engineering, National Yunlin University of Science and Technology, Douliou 64002, Taiwan; 2 Department of Physics, National Chung Cheng University, Chia-Yi 62102, Taiwan; E-Mail: phyjlt@ccu.edu.tw

**Keywords:** photonic crystal fiber (PCF), interferometer, refractive index (RI), wavelength shift, fringe period, fringe visibility, sensitivity, 42.81.Pa, 07.60.Ly, 78.20.Ci, 07.60.Vg

## Abstract

We report on a refractive index sensor using a photonic crystal fiber (PCF) interferometer which was realized by fusion splicing a short section of PCF (Blaze Photonics, LMA-10) between two standard single mode fibers. The fully collapsed air holes of the PCF at the spice regions allow the coupling of PCF core and cladding modes that makes a Mach-Zehnder interferometer. The transmission spectrum exhibits sinusoidal interference pattern which shifts differently when the cladding/core surface of the PCF is immersed with different RI of the surrounding medium. Experimental results using wavelength-shift interrogation for sensing different concentrations of sucrose solution show that a resolution of 1.62 × 10^−4^–8.88 × 10^−4^ RIU or 1.02 × 10^−4^–9.04 × 10^−4^ RIU (sensing length for 3.50 or 5.00 cm, respectively) was achieved for refractive indices in the range of 1.333 to 1.422, suggesting that the PCF interferometer are attractive for chemical, biological, biochemical sensing with aqueous solutions, as well as for civil engineering and environmental monitoring applications.

## 1. Introduction

Optical refractometer sensors based on waveguide technology are promising and attractive for chemical and biotechnological applications. The advantages of this type of sensor are their relatively simple construction, compactness, low cost, ease of use, immunity to electromagnetic interference, and high sensitivity to the external refractive index (RI) [1–10]. Recently, several types of optical waveguide sensors or optical fiber-based refractometer have been proposed, including long-period fiber gratings, Fabry-Perot interferometers and Mach-Zehnder interferometers. The interferometers utilizing a pair of collapsed zones separated by a few centimeters are suitable for sensing applications for they offer many advantages such as robustness, low insertion losses and relatively simple fabrication. However, they suffer from a serious disadvantage: high sensitivity to the environmental temperature. Photonic crystal fiber (PCF) has the potential to compensate for this drawback because its dispersion properties are strongly dependent on the air–hole geometry [11]. This fiber is typically fabricated using the stack-and-draw technique [12], making it possible to produce a wide variety of geometries. Thus, other than external RI, the operation principle of the PCF interferometers built via micro-holes collapsing is based on the excitation and recombination of two modes. The first collapsing point induces the coupling of a fraction of the incident light propagating in the core mode to the cladding mode, and the second one performs the opposite function, generating the interference of light propagating in different optical paths. PCF-like interferometers and assembly have been used for measuring physical, chemical, and biochemical measurands such as strain, high temperature (up to 1,000 °C), hydrostatic pressure, curvature, biofilm, and chemical vapor [8,10,13–20].

In this paper, we present a PCF interferometer made by fusion splicing a piece of PCF between two standard single mode fibers with a fusion splicer. We report on the investigation and analysis of this class of novel PCF interferometers for refractive index sensing. The ability of the PCF interferometers, with five different sensing lengths (1.34, 2.67, 3.50, 4.40, and 5.00 cm), as a high sensitive RI sensor will be exploited. The unique sensing features of these spectral filters are particularly suitable for a wide variety of applications in sensor systems.

## 2. Device Fabrication and Experiment

### 2.1. Principle of Interferometer

According to the interference theory, the value of the fringe period (Λ) of the Mach-Zehnder interferometer at any *λ* can be expressed as follows [13,14]:

(1) $$\Lambda =\frac{2\pi \lambda }{(\beta_{1}-\beta_{2})\cdot L}$$

where *λ* is the central wavelength of the light source; *β*_1_ and *β*_2_ are propagation constants of the modes involved in the interference, and *L* is the length of the interferometer. The fringe period is inversely proportional to propagation constant deviation Δ*β = β*_1_ − *β*_2_ and the interferometer length *L*.

### 2.2. Fabrication of PCF Interferometers

**Figure 1.**
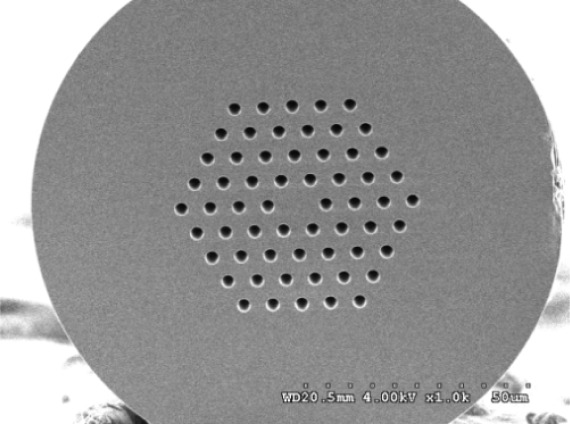
SEM image for the cross-section of the LMA-PCF used to fabricate the PCF interferometer.

**Figure 2.**
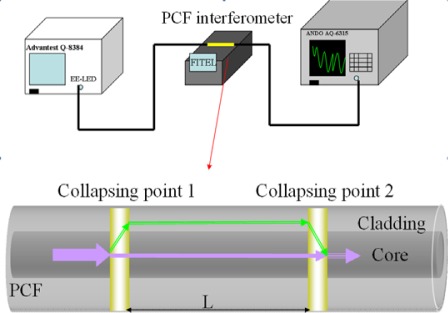
The top figure shows the experimental setup for the fabrication of a PCF interferometer. The bottom diagram is a PCF interferometer built via microhole collapsing and L is the sensing length (between two collapsed zones).

The interferometer was fabricated by using a LMA-PCF fiber (Blaze Photonics, LMA-10) which was designed to have a large effective mode field area (∼40 μm). It has an outside diameter of 125 μm, a core diameter of 10 μm, a hole diameter of 3.0 μm, and a hole pitch of 7.0 μm. Figure 1 shows a scanning electron micrograph (SEM) of the cleaved face of a LMA-PCF. A piece of PCF was fusion spliced with two conventional optical fibers (Corning SMF-28), head on head and one on each side using a fusion splicer (Fitel S175). It was found that the splice loss was about 1 dB. Figure 2 shows the experimental setup for fabricating the PCF interferometer. The PCF interferometer or Mach-Zehnder-like interferometer was constructed by making two collapsing points in a PCF at localized regions with a fusion splicer. For the above five different sensing lengths of PCF interferometers, the diameters of both collapsing points were in the range of 120 to 230 μm. A high fringe contrast suitable for making PCF interferometer could be obtained by tuning the arc power and arc time and the optimum parameters were found to be 128 mA and 350 ms, respectively. During the course of fabrication, the spectrum of the PCF interferometer was monitored with a broadband light source (center wavelength 1,550 nm) and an optical spectrum analyzer (OSA, ANDO AQ6315A).

### 2.3. Experiment

**Figure 3.**
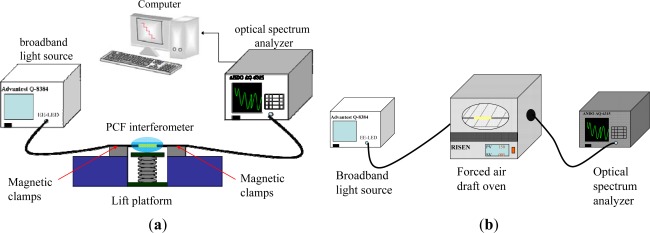
Schematic of the experimental setup using the PCF interferometers for (**a**) refractive index measurements; (**b**) temperature sensing measurements.

Figure 3 illustrates the experimental setup for refractive index and temperature sensing measurements with the PCF interferometer. The fiber-optic sensing system used to measure the transmission spectrum of the sensor consists of a broadband light source (λ = 1,550 nm), a sensing PCF interferometer, an OSA (ANDO AQ6315A), and a computer for data recording.

The characteristic measurements were conducted for interferometers associated with five different sensing lengths (*i.e.*, the length between two collapsed zones, *L* = 1.34, 2.67, 3.50, 4.40, and 5.00 cm). We fabricated three replicates for each length of interferometer and there are totally 15 interferometers in this study. The interference spectra contain the information of wavelength, transmission loss, and fringe period. The distance between any two consecutive peak fringes is determined as a fringe period. The fringe visibility was calculated for each PCF interferometer as follows [14]:

(2) $$\frac{I_{\text{max}}-I_{\text{min}}}{I_{\text{max}}+I_{\text{min}}}\%$$

where *I_max_* and *I_min_* are the maximum and minimum intensity values of corresponding interference spectrum, respectively.

**Figure 4.**
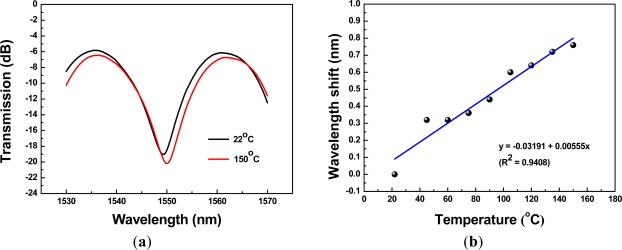
(**a**) The transmission spectra of a 2.67-cm PCF interferometers when heated from 22 °C to 150 °C; (**b**) The relationship between wavelength and temperature for this 2.67-cm PCF interferometers (temperature sensitivity = 5.55 pm/°C).

The RI measurements were performed using the above 15 interferometers and the refractive indices were in the range of 1.333 to 1.422. The interference spectra and the relationship between wavelength shift and refractive index were used to characterize the PCF Mach-Zehnder interferometers. During the course of data measurement, the magnetic clamps and a lift platform were used to keep the PCF interferometers straight lines without the bending effect [see Figure 3(a)]. We have maintained the condition of strain, bending, and temperature quite stable in the laboratory so that they were not the concerns of this work. For precise measurement, we kept the experimental setup and sample materials at a constant ambient temperature (within 1 °C fluctuation). As mentioned in the introduction [11], we also conducted temperature sensing experiment to prove that the PCF interferometers have the potential to compensate high sensitivity to environmental temperature. For example, a 2.67-cm PCF interferometer was heated from 22 °C to 150 °C using a forced air draft oven [see Figure 3(b)]. The transmission spectra of this PCF interferometer are shown in Figure 4(a). The relationship between wavelength shift and temperature was shown in Figure 4(b). The corresponding sensing slope is the temperature sensitivity of a PCF interferometer [temperature sensitivity = 5.55 pm/°C, see Figure 4(b)]. We have obtained the temperature sensitivity of all five different lengths of PCF interferometers was in the range of 5.55–8.29 pm/°C. As the environmental temperature change is about 50 °C, the corresponding wavelength shifts of PCF interferometers are less than 0.4 nm. Thus, the PCF interferometers have the potential to compensate high sensitivity to general environmental temperature.

## 3. Results and Discussion

### 3.1. PCF Interferometer Characteristics

**Figure 5.**
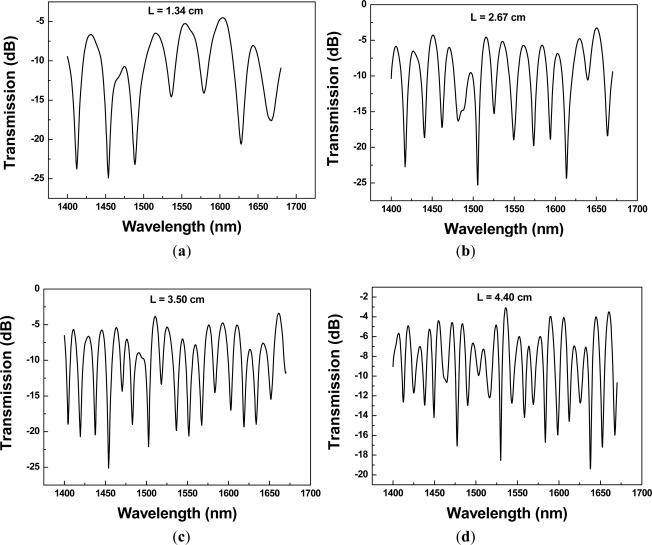

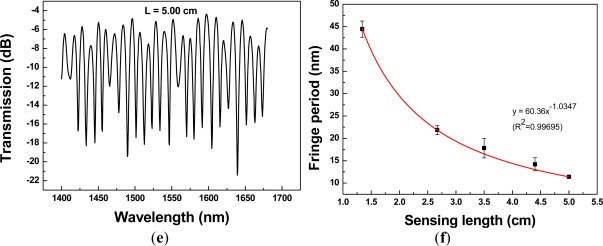
(**a–e**) The transmission spectra of the PCF interferometers with several sensing lengths; (**f**) Plot of fringe period as a function of sensing length.

**Figure 6.**
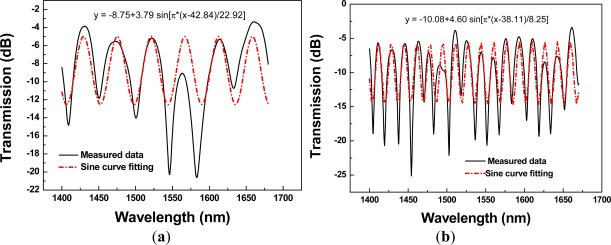
Plot of transmission spectra of measured data and sine curve fitting using PCF interferometers with (**a**) L = 1.34 cm and (**b**) L = 3.50 cm, respectively.

**Figure 7.**
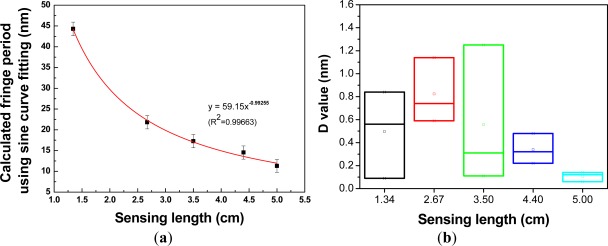
(**a**) Plot of calculated fringe periods from sine curve fitting *versus* sensing length; (**b**) Plot of D value *versus* sensing length.

The transmission spectra of a PCF interferometer with different sensing lengths are shown in Figure 5(a–e). High uniform interference fringes were observed over the available spectral range, and the average fringe periods became larger with the decreasing sensing length. The average fringe periods as a function of sensing length, obtained using a linear regression analysis, was determined to be Λ = 60.36×*L*^−1.0347^ [*R*^2^ = 0.99695, see Figure 5(f)], indicative of the effect of the Mach-Zehnder interferometer. The fringe visibility was calculated for the 15 PCF interferometers and they were in the range of 62.16–89.39%. Since we used the same type of PCF (Blaze Photonics, LMA-10) and the propagation constant deviation involved in the interference could be assumed the same; thus, based on the Figure 5(f), the fringe period of the interferometer was almost inversely proportional to the interferometer length (*R*^2^ = 0.996995). In addition, we compared the measured fringe period and the one from a sine curve fitting for each interferometer. Figure 6(a,b) shows the transmission spectra of measured data and sinusoidal wave using a PCF interferometer with length = 1.34 cm and 3.50 cm, respectively. The sinusoidal curve is given by *y* = *A_o_* + *A* sin (*ωx* - *φ*); where *y* is the transmission loss, *x* represents the wavelength, and other parameters are the regression coefficients. Figure 7(a) illustrates the plot of calculated fringe period from sine curve fitting *versus* sensing length. A correlation was found that the calculated fringe period based on sine curve fitting was almost inversely proportional to the sensing length—Λ = 59.15×*L*^−0.99255^ (*R*^2^ = 0.99663). The absolute difference value between average measured fringe period and the one from sine curve fitting is calculated as follows:

(3) D=| average measured fringe period − calculated fringe period from sine curve fringe |

The box plot (or whisker graph) is a statistical way of graphically depicting groups of numerical data through their five-number summaries: maximum, minimum, median, upper quartile, and lower quartile. In addition, it could be even used to explore outliers [21]. Figure 7(b) displays the box plot for the *D* value *versus* sensing length. The results show that the PCF interferometer has smaller *D* values, 0.48 nm and 0.14 nm, when sensing lengths are 4.40 cm and 5.00 cm, respectively.

### 3.2. Refractive Index Measurement

**Figure 8.**
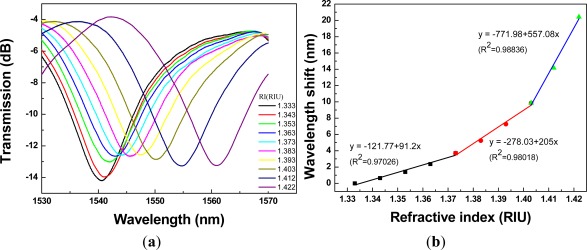
A 1.34-cm PCF interferometer with: (**a**) the transmission spectra for sensing different refractive indices of the sucrose solution (RI = 1.333 – 1.422); (**b**) the relationship between wavelength shift and refractive index (resonance wavelength = 1,540.60 nm).

The ability of the PCF interferometer to detect changes in the surrounding RI was studied. The control of surrounding RI was through the use of sucrose solutions with various concentrations. Figure 8(a) shows the plot of transmission spectra of a 1.34-cm PCF interferometer with resonance wavelength = 1,540.60 nm, for sensing different refractive indices of the sucrose solution (RI =1.333 − 1.422). Since the relationship between wavelength shift and refractive index is nonlinear; thus, we quantified the above relationship using three-stage refractive index area, where the three-stage refractive index area are as follows: first refractive index area (RI = 1.333 − 1.373); second refractive index area (RI = 1.373 − 1.403); and third refractive index area (RI = 1.403 − 1.422).

**Figure 9.**
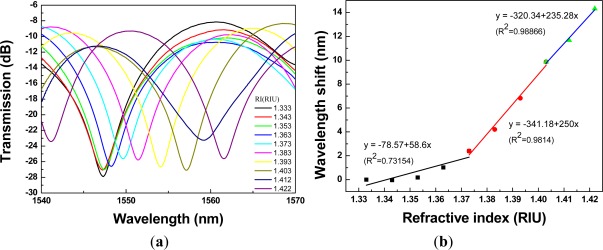
A 2.67-cm PCF interferometer with: (**a**) the transmission spectra for sensing different refractive indices of the sucrose solution (RI = 1.333 – 1.422); (**b**) the relationship between wavelength shift and refractive index (resonance wavelength = 1,547.26 nm).

**Figure 10.**
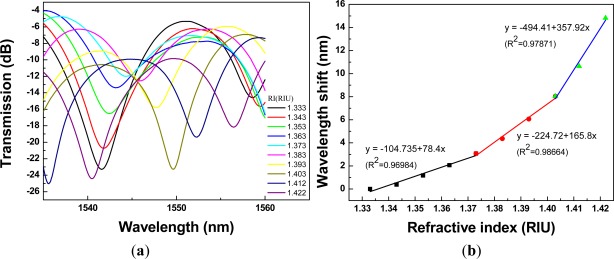
A 3.50-cm PCF interferometer with: (**a**) the transmission spectra for sensing different refractive indices of the sucrose solution (RI =1.333 – 1.422); (**b**) the relationship between wavelength shift and refractive index (resonance wavelength = 1,541.56 nm).

**Figure 11.**
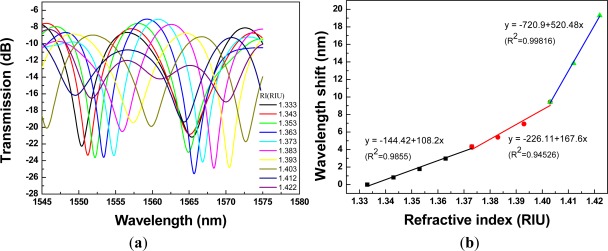
A 4.40-cm PCF interferometer with: (**a**) the transmission spectra for sensing different refractive indices of the sucrose solution (RI =1.333 – 1.422); (**b**) the relationship between wavelength shift *versus* refractive index (resonance wavelength = 1,550.42 nm).

**Figure 12.**
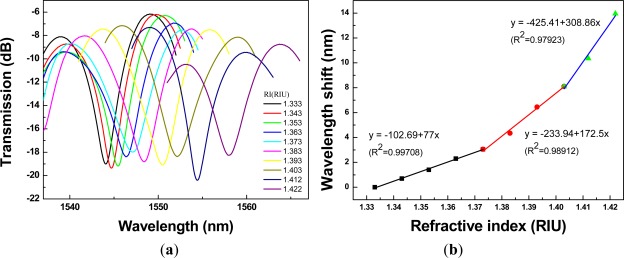
A 5.00-cm PCF interferometer with: (**a**) the transmission spectra for sensing different refractive indices of the sucrose solution (RI =1 .333 – 1.422); (**b**) the relationship between wavelength shift *versus* refractive index (resonance wavelength = 1,544.05 nm).

Regarding Figures 8(b) to 12(b), we performed 15 linear regression analyses using the above three-stage RI areas. Other than one regression analysis, the R^2^ values were greater than 0.94. Thus, the relationship between wavelength shift and RI for PCF interferometers was assessed as a three-stage linear function and the boundary of the three-stage linear regression was determined. The graph of wavelength shift *versus* refractive index and three-stage linear regression models were plotted in Figure 8(b). We also conducted the RI experiment for the different four lengths of PCF interferometers and the results were shown in Figures 9 to 12 for sensing length *L* = 2.67 m, 3.50 cm, 4.40 cm, and 5.00 cm, respectively.

**Figure 13.**
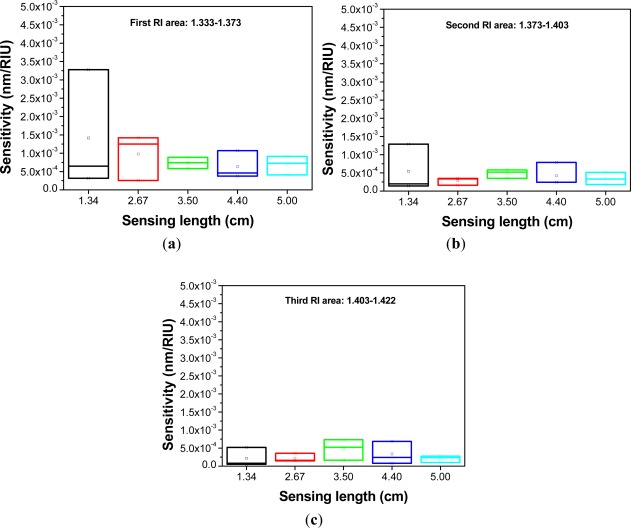
Plot of the relationship between sensitivity and sensing length for PCF interferometer at (**a**) first refractive index area (RI = 1.333 – 1.370); (**b**) second refractive index area (RI = 1.373 – 1.403); and **(c)** third refractive index area (RI = 1.403 – 1.422).

A red shift in the resonance wavelength was observed with the increase of RI as shown in Figures 8 to 12. The graphs display transmission spectra *versus* air and RI for different concentrations of sucrose solution using a PCF interferometer. For example, Figure 12(b) shows a linear fit (R^2^ = 0.9971, 0.9891, and 0.9792) to the plot of the wavelength shift *vs.* RI of the sucrose solution. For the RI sensitivity of a 5.00-cm PCF interferometer was determined by the slopes of three-stage linear fits and it had 309 nm/RIU, 251 nm/RIU, and 326 nm/RIU in the RI range of 1.333–1.373, 1.373–1.403, and 1.403–1.422, respectively. We have collected and analyzed the data considering different sensing lengths in the PCF interferometer. Figure 13(a–c) shows the box plots of interferometer resolution (sensor resolution = 3*σ*/*m*, *σ* = standard deviation of sensor response in measuring the blank sample, *m* = slope) *versus* the sensing length (1.34–5.0 cm) for three-stage RI areas. Studies presented here demonstrate that these PCF interferometers (sensing length = 1.34, 2.67, 3.50, 4.40, or 5.00 cm) could provide a sensor resolution of 3.28 × 10^−3^–5.20 × 10^−5^ RIU for refractive indices in the range of 1.333 to 1.422. For example, the resolution of PCF interferometer was as low as in the range of 1.62 × 10^−4^–8.88 × 10^−4^ RIU or 1.02 × 10^−4^–9.04 × 10^−4^ RIU when sensing length is equal to 3.50 cm or 5.00 cm, respectively. Based on Figure 13(a–c), the five different lengths of PCF interferometers were evaluated and the effect of sensor length was discussed about the sensor sensitivity (nm/RIU) and sensitivity variation (sensitivity range). The PCF interferometers with sensing length 3.50 cm and 5.00 cm had both better sensor sensitivity and smaller sensitivity variation than those of other PCF interferometers. Thus, the 3.00-cm and 5.00-cm PCF interferometers possessed comparable performance for the resolution in the range of 1.62 × 10^−4^–8.88 × 10^−4^ RIU and 1.02 × 10^−4^–9.04 × 10^−4^ RIU, respectively. In addition, the 3.00-cm and 5.00-cm PCF interferometers exhibited relatively small sensitivity variation (less than 5.71 nm/RIU and 5.05 nm/RIU, respectively). However, if the 5.00-cm PCF interferometer is used, the bending effect and package technique should be noticed.

## 4. Conclusions

We have successfully demonstrated the feasibility of fabricating a class of highly sensitive refractive-index sensor based on the PCF interferometer that was realized by fusion splicing a short section of PCF between two standard single mode fibers (Corning SMF-28) using a Fitel-S175 splicer. The interferometer was completed by making two collapsing points in a PCF at localized regions with a fusion splicer. The high uniform interference fringes over the available spectral range (about 1,400–1,675 nm) could be obtained. The realization of the sensor is through the measurement of the transmission spectrum of the PCF interferometer. A red shift in the resonance wavelength was observed with the increase of RI. We demonstrate the sensor resolution for different lengths of PCF interferometers by transmission spectrum interrogation, and the findings indicating that a resolution as low as 1.62 × 10^−4^–8.88 × 10^−4^ RIU or 1.02 × 10^−4^–9.04 × 10^−4^ RIU (for sensing length = 3.50 cm or 5.00 cm, respectively) can be achieved for refractive indices in the range of 1.333 to 1.422. The 3.00-cm and 5.00-cm PCF interferometers possessed comparable performance for the resolution in the range of 1.62 × 10^−4^–8.88 × 10^−4^ RIU and 1.02 × 10^−4^–9.04 × 10^−4^ RIU, respectively. Furthermore, the 3.00-cm and 5.00-cm PCF interferometers exhibited relatively small sensitivity variation (less than 5.71 nm/RIU and 5.05 nm/RIU, respectively). Such a highly sensitive PCF interferometer is attractive for chemical, biological, biochemical sensing with aqueous solutions, as well as for civil engineering and environmental monitoring applications.

## References

[1] Cheng S.F., Chau L.K. (2003). Colloidal Gold-Modified Optical Fiber for Chemical and Biochemical Sensing. Anal. Chem.

[2] Chau L.K., Lin Y.F., Cheng S.F., Lin T.J. (2006). Fiber-Optic Chemical and Biochemical Probes Based on Localized Surface Plasmon Resonance. Sens. Actuat. B.

[3] Frazão O., Viegas J., Caldas P., Santos J.L., Araújo F.M., Ferreira L.A., Farah F. (2007). All-Fiber Mach-Zehnder Curvature Sensor Based on Multimode Interference Combined with a Long-Period Grating. Opt. Lett.

[4] Tang J.-L., Wang J.-N. (2008). Chemical Sensing Sensitivity of Long-Period Grating Sensor Enhanced by Colloidal Gold Nanoparticles. Sensors.

[5] Chen C.-H., Chao T.-C., Li W.-Y., Shen W.-C., Cheng C.-W., Tang J.-L., Chau L.-K., Wu W.-T. (2010). Novel D-Type Fiber Optic Localized Plasmon Resonance Sensor Realized by Femtosecond Laser Engraving. J. Laser Micro/Nanoeng.

[6] Chen C.-H., Tsao T.-C., Tang J.-L., Wu W.-T. (2010). A Multi-D-Shaped Optical Fiber for Refractive Index Sensing. Sensors.

[7] Jha R., Villatoro J., Badenes G. (2008). Ultrastable in Reflection Photonic Crystal Fiber Modal Interferometer for Accurate Refractive Index Sensing. Appl. Phy. Lett.

[8] Rindorf L., Bang O. (2008). Sensitivity of Photonic Crystal Fiber Grating Sensors: Biosensing, Refractive Index, Strain, and Temperature Sensing. J. Opt. Soc. Am. B.

[9] Nunes P.S., Mortensen N.A., Kutter J.P., Mogensen K.B. (2010). Refractive Index Sensor Based on a 1D Photonic Crystal in a Microfluidic Channel. Sensors.

[10] Jha R., Villatoro J., Badenes G., Pruneri V. (2009). Refractometry Based on a Photonic Crystal Fiber Interferometer. Opt. Lett.

[11] Birks T.A., Knight J.C., Russel P.S.J. (1997). Endlessly Single-Mode Photonic Crystal Fiber. Opt. Lett.

[12] Knight J.C., Birks T.A., Russell P.S.J., Atkin D.M. (1996). All-Silica Single-Mode Optical Fiber with Photonic Crystal Cladding. Opt. Lett.

[13] Choi H.Y., Kim M.J., Lee B.H. (2007). All-fiber Mach-Zehnder Type Interferometers Formed in Photonic Crystal Fiber. Opt. Exp.

[14] Villatoro J., Minkovich V.P., Pruneri V., Badenes G. (2007). Simple All-Microstructured-Optical-Fiber Interferometer Built Via Fusion Splicing. Opt. Exp.

[15] Fávero F.C., Quintero S.M.M., Martelli C., Braga A.M., Silva V.V., Carvalho I.C.S., Llerena R.W.A., Valente L.C.G. (2010). Hydrostatic Pressure Sensing with High Birefringence Photonic Crystal Fibers. Sensors.

[16] Villatoro J., Kreuzer M.P., Jha R., Minkovich V.P., Finazzi V., Badenes G., Pruneri V. (2009). Photonic Crystal Fiber Interferometer for Chemical Vapor Detection with High Sensitivity. Opt. Exp.

[17] Silva S.F.O., Santos J.L., Kobelke J., Schuster K., Frazão O. (2011). Simultaneous Measurement of Three Parameters Using an All-Fiber Mach-Zehnder Interferometer Based on Suspended Twin-Core Fibers. Opt. Eng.

[18] Chen W., Lou S., Wang L., Jian S. (2010). Ring-Core Photonic Crystal Fiber Interferometer for Strain Measurement. Opt. Eng.

[19] Coviello G., Finazzi V., Villatoro J., Pruneri V. (2009). Thermally Stabilized PCF-Based Sensor for Temperature Measurements up to 1000 °C. Opt. Exp.

[20] Villatoro J., Finazzi V., Minkovich V.P., Pruneri V., Badenes G. (2007). Temperature-Insensitive Photonic Crystal Fiber Interferometer for Absolute Strain Sensing. Appl. Phy. Lett.

[21] Hogg R.V., Tanis E.A. (1993). Probability and Statistical Inference.

